# Correction: Tallapaneni et al. Growth Factor Loaded Thermo-Responsive Injectable Hydrogel for Enhancing Diabetic Wound Healing. *Gels* 2023, *9*, 27

**DOI:** 10.3390/gels12050421

**Published:** 2026-05-12

**Authors:** Vyshnavi Tallapaneni, Lavanya Mude, Divya Pamu, Vasanth Raj Palanimuthu, Sai Varshini Magham, Veera Venkata Satyanarayana Reddy Karri, Madhukiran Parvathaneni

**Affiliations:** 1 Department of Pharmaceutics, JSS College of Pharmacy, JSS Academy of Higher Education & Research, Ooty 643001, India; vyshutallapaneni@gmail.com (V.T.); lavanyamude4h@gmail.com (L.M.); pamudivya143@gmail.com (D.P.); 2 Department of Pharmaceutical Biotechnology, JSS College of Pharmacy, JSS Academy of Higher Education & Research, Ooty 643001, India; vasanth@jssuni.edu.in; 3 Department of Pharmacology, JSS College of Pharmacy, JSS Academy of Higher Education & Research, Ooty 643001, India; varshinimagham1998@gmail.com; 4 Department of Biotechnology, Harrisburg University of Science & Technology, 326 Market Street, Harrisburg, PA 17101, USA; 5 Arni Medica, 4475 South Clinton Ave, Suite 230, South Plainfield, NJ 07080, USA; 6 CRC Pharma LLC, 333 Littleton Road, Parsippany, NJ 07054, USA

In the original publication [1], there were duplications in the subfigures of Figure 12. The authors have conducted new experiments, and the corrected Figure 12 is shown below. The authors state that the scientific conclusions are unaffected. This correction was approved by the Academic Editor. The original publication has also been updated.

## Figures and Tables

**Figure 12:**
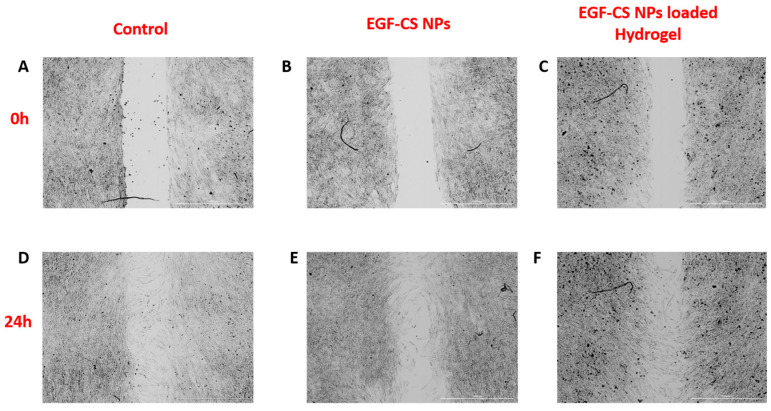
Cell migration in 3T3-L1 with or without (control) treatment for 24 h. Cell migration was evaluated in untreated (control) and treated groups over 24 h. A uniform scratch was created in confluent monolayers, and images were captured at 0 h and 24 h. At 0 h, all groups (control, EGF-CS NPs, and EGF-CS NPs-loaded hydrogel) showed the initial wound area immediately after scratching. At 24 h, wound closure is observed in control cells and cells treated with EGF-CS NPs and EGF-CS NPs-loaded hydrogel. Representative images are shown for: (**A**,**D**): control (0 h and 24 h), (**B**,**E**): EGF-CS NPs-treated cells (0 h and 24 h), and (**C**,**F**): EGF-CS NPs-loaded hydrogel-treated cells (0 h and 24 h). The extent of wound closure reflects the migratory capacity of the cells under different treatment conditions.
